# Supplementary material for: Risk factors for sacrococcygeal pilonidal sinus: a systematic review and meta-analysis supplemented by genetic causal assessment
Source: Front Surg. 2026 Jan 7;12:1718589. doi: 10.3389/fsurg.2025.1718589 (PMC12819706; doi:10.3389/fsurg.2025.1718589)
Supplement: Supplementary file 2 [file Datasheet2.zip › Supplementary Data 2/MR_pipeline_p5e-6/leaveone_plots/ebi-a-GCST006094.finngen_R12_L12_PILONIDALCYST.pdf]

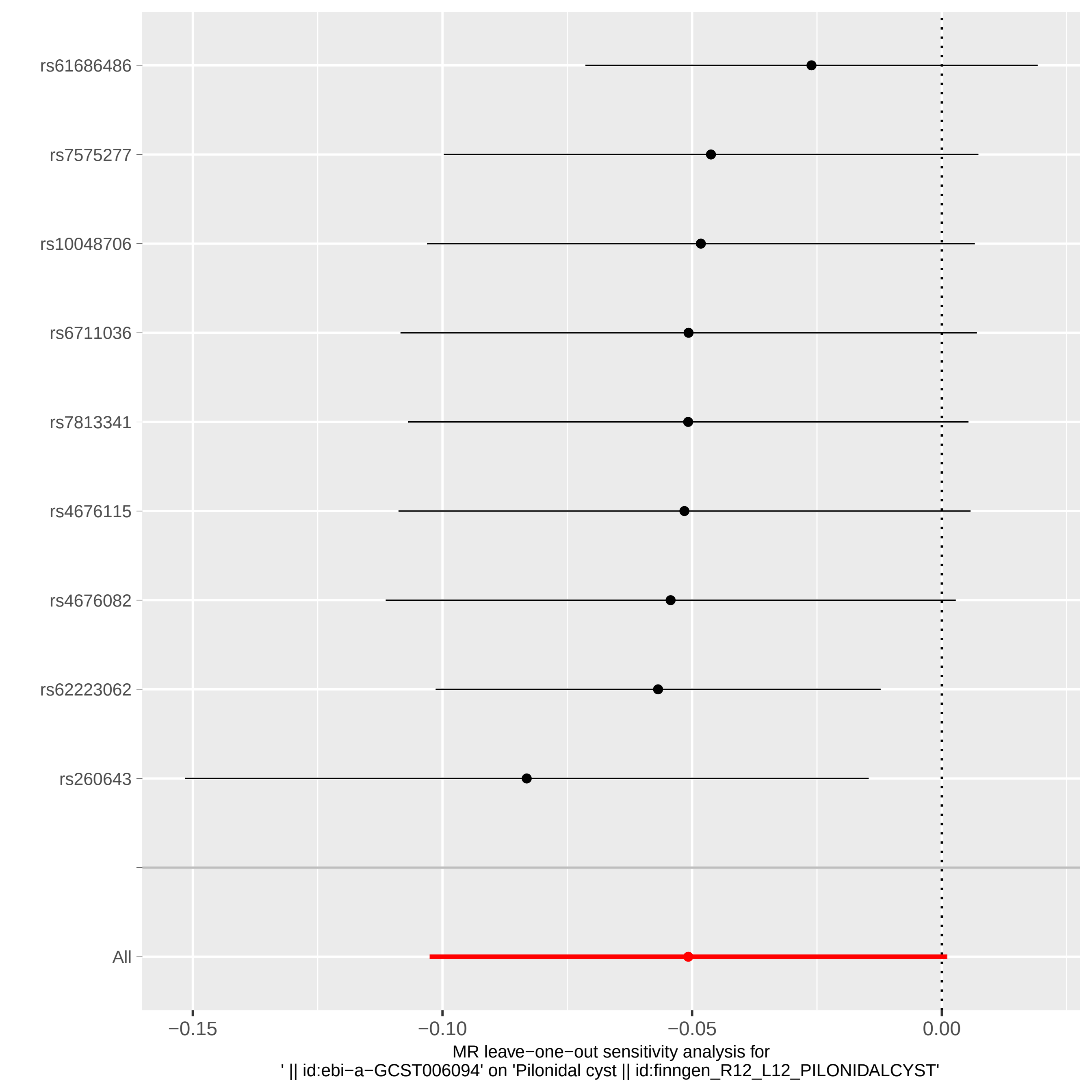
rs61686486
rs7575277
rs10048706
rs6711036
rs7813341
rs4676115
rs4676082
rs62223062
rs260643
All
−0.15
−0.10
−0.05
0.00
MR leave−one−out sensitivity analysis for
' || id:ebi−a−GCST006094' on 'Pilonidal cyst || id:finngen_R12_L12_PILONIDALCYST'
